# Choline supplements: An update

**DOI:** 10.3389/fendo.2023.1148166

**Published:** 2023-03-07

**Authors:** Urna Kansakar, Valentina Trimarco, Pasquale Mone, Fahimeh Varzideh, Angela Lombardi, Gaetano Santulli

**Affiliations:** ^1^ Department of Medicine, Division of Cardiology, Einstein Institute for Aging Research, Montefiore Health System, New York, NY, United States; ^2^ University of Naples “Federico II”, Naples, Italy; ^3^ ASL Avellino, Montefiore Health System, New York, NY, United States; ^4^ Department of Microbiology and Immunology, Montefiore Health System, New York, NY, United States; ^5^ Department of Molecular Pharmacology, Einstein-Sinai Diabetes Research Center (ES-DRC), Montefiore Health System, New York, NY, United States

**Keywords:** choline, choline alfoscerate, choline bitartrate, choline supplementation, cognitive dysfunction, GPC, lecithin, supplements

## Abstract

In this comprehensive review, we examine the main preclinical and clinical investigations assessing the effects of different forms of choline supplementation currently available, including choline alfoscerate (C_8_H_20_NO_6_P), also known as alpha-glycerophosphocholine (α-GPC, or GPC), choline bitartrate, lecithin, and citicoline, which are cholinergic compounds and precursors of acetylcholine. Extensively used as food supplements, they have been shown to represent an effective strategy for boosting memory and enhancing cognitive function.

## Introduction

Choline is an important nutrient essential for proper functioning of liver, muscle, and brain (1–5). It is a main constituent of cell and organelle membranes and plays a vital role in numerous physiological processes including signal transduction, DNA and histone methylation, and nerve myelination (6, 7). Choline is a precursor of different metabolites including the neurotransmitter acetylcholine (ACh), the membrane phospholipids phosphatidylcholine (PC) and sphingomyelin, and the methyl donor betaine.

Choline can be obtained from the diet and *via de novo* biosynthesis from the methylation of phosphatidylethanolamine (PE) to PC (6, 8). The demand for choline increases particularly during pregnancy inasmuch as it is important for placental function, fetal growth, and brain development (7). Choline deficiency can cause serious medical conditions such as premature birth, cystic fibrosis, and hepato-steatosis. Therefore, a sufficient choline intake is necessary for growth and homeostasis.

The US Food and Drug Administration (FDA) identified choline as an essential nutrient in 1998. The National Academy of Medicine (NAM) of the USA and the European Food Safety Authority (EFSA) both specified adequate intake (AI) values for choline. Of course, age, sex, life conditions (pregnancy, breastfeeding), and genetic polymorphisms represent central factors in determining AI (9). In 2016, the European Food Safety Authority (EFSA) set an AI of 400 mg/day for all healthy adults. Similarly, the AIs for pregnant and lactating women are 480 mg/day for and 520 mg/day respectively. The US Institute of Medicine (IOM) has a slightly different choline AIs set for nonpregnant, pregnant, and lactating women: 425 mg/day, 450 mg/day, and 550 mg/day, respectively. Low AIs were set for infants of various ages: AI recommendations for infants 0–6 months are 125 mg choline/day whereas for infants 7–12 months are 150 mg choline/day. These AI values are set according to choline concentrations in human milk (160 mg/L) and estimated average volume of human milk intake (0.78 L/day) for a whole group of infants (aged 0–6 months) with a default body weight of 7 kg (approximately 18 mg/kg), and extrapolation for default body weight (aged 7-12 months) (10, 11). Plasma choline concentrations are three times higher in newborn infants than in their mothers as human milk is rich in choline (12–16).

## Choline alfoscerate

In addition to choline intake from food, there are several forms of choline supplementation currently available (2). Choline alfoscerate (C_8_H_20_NO_6_P), also known as alpha-glycerophosphocholine (α-GPC, or GPC), is a cholinergic compound and ACh precursor extensively used as a food supplement. Its molecular weight is 257.22 g/mol. GPC is considered one of the most used sources of choline due to its high choline content (41% of choline by weight) and its ability to cross the blood-brain barrier. The content of choline and GPC in common foods is reported in **Table 1**.

**Table 1:** Choline and GPC content in common foods (mg choline moiety/100 g of food) according to the US Department of Agriculture (USDA); the NDB (Nutrient DataBase) identifier is a five-digit numerical code used by the USDA for standard reference.

NDB No.	Description	Free Choline	GPC
*35180*	Fish, steelhead trout, dried, flesh (Shoshone Bannock)	15.0	190.0
*35190*	Fish, salmon, red (sockeye), smoked (Alaska Native)	46.0	130.0
*35153*	Fish salmon, king (chinook), raw (Alaska Native)	20.0	50.0
*35151*	Fish, salmon, sockeye (red), raw (Alaska Native)	20.0	53.0
*35169*	Fish, sheefish, dried (Alaska Native)	12.0	74.0
*35055*	Seal, bearded (oogruk), meat, air-dried (Alaska Native)	17.0	52.0
*35152*	Fish, salmon, chum, raw (Alaska Native)	23.0	41.0
*15237*	Fish, salmon, Atlantic, farmed, cooked, dry heat	7.8	41.0
*15236*	Fish, salmon, Atlantic, farmed, raw	9.9	43.0
*19120*	Candies, milk chocolate	9.1	22.0
*01079*	Milk, reduced fat fluid, 2% milk fat, with added vitamin A	2.8	10.0
*01117*	Yogurt, plain, low fat, 12 g protein per 8 oz	2.3	9.1
*08231*	Cereals, Quaker, Oat Bran, Quaker/Mother’s Oat Bran, dry	4.4	33.0
*98032*	Candies, milk chocolate pieces, sugar coated	9.6	22
*98034*	Frozen yogurts, vanilla, fat free	3.7	13.0
*18375*	Leavening agents, yeast, baker’s, active dry	6.1	16.0
*18927*	Crackers, cheese, sandwich-type with cheese filling	6.7	15.0
*18452*	Cake, snack cakes, cupcakes, chocolate, with frosting, low- fat	5.0	10.0
*01046*	Cheese food, pasteurized process, American, without sodium phosphate	7.9	14.0
*98031*	Candies, milk chocolate coated wafer bars	7.9	16.0

After oral administration, GPC can be readily metabolized to PC, the active form of choline that is able to increase the release of the neurotransmitter ACh (17, 18) and brain-derived neurotrophic factor (BDNF) (19, 20). GPC enhances memory and cognitive function and is well-known to be effective in the treatment of several neurodegenerative and vascular diseases such as Alzheimer’s disease and dementia (21–23). GPC has been shown to be more effective when combined with cholinesterase inhibitors (24, 25). Numerous studies have identified the favorable effects of GPC in the treatment of the sequelae of cerebrovascular accidents (26–28).

Nevertheless, GPC can be a friend or a foe depending on the doses and length of its administration. Uncovering a safe therapeutic window is essential to prevent adverse reactions.

## Preclinical studies

GPC has been shown to exhibit a favorable action in experimental models of the aging brain as well as in a rat model of pilocarpine-induced seizure (29, 30), and to promote neuronal differentiation in a rat model of noise-restraint stress (29). *In vitro* assays performed in the SH-SY5Y human cell line have revealed that this cholinergic compound antagonizes neurotoxicity triggered by the fragment *Aβ (* 25–35) of the Alzheimer’s amyloid β-peptide and attenuates the Aβ-induced phosphorylation of the Tau protein (31), by sustaining the expression level of synaptic vesicle proteins, such as synaptophysin (32–34). GPC was also shown to increase hippocampal neurogenesis, providing protection against seizure-induced neuronal death and cognitive impairment (26) and to antagonize scopolamine-induced amnesia enhancing hippocampal cholinergic transmission, suggesting that the behavioral effects of GPC could be related to its property to increase hippocampal synthesis and release of ACh (35–38).

Although GPC does not seem to be directly involved in the modulation of inflammatory responses (39), it has been shown to improve mitochondrial function and to reduce oxidative and nitrosative stress (40).

Chronic treatment of aged rats with GPC restored the number of muscarinic M_1_ receptors to levels found in the striatum and hippocampus from young animals (41). In young but not old rats, GPC significantly potentiated K^+^-stimulated intra-synaptosomal Ca^2+^ oscillations in purified synaptosomes derived from the hippocampus (17). Repeated injections of GPC significantly increased basal formation of [^3^H]inositol monophosphate in hippocampal, cortical, and striatal slices of male rats (42). Consistently, GPC potentiated receptor-stimulated phosphatidylinositol hydrolysis in cortical synapto-neurosomes (17).

In a model of acute cerebral ischemia in rats, GPC increased the tolerance of neurons to ischemic damage and slowed the execution of the cell death program (43). Consistent with these findings, *in vitro* assays in astroglial cell cultures have shown that GPC increases proliferation (44).

## Clinical investigations

Cholinergic precursors have represented one of the first approaches attempting to relief cognitive impairment in dementia-related disorders. However, controlled clinical trials failed to show significant improvements with choline or PC, choline-containing phospholipids, alone or in association with cholinesterase inhibitors (tacrine plus choline, or physostigmine plus choline) (44, 45). Luckily, the lack of clinical benefits obtained with choline or lecithin are not shared by other phospholipids involved in choline biosynthetic pathways, including GPC and citicoline **(**cytidine 5′-diphosphocholine, also known as CDP-choline), which are able to increase ACh content and release (44, 46).

A study in male young adults demonstrated that the ingestion of 1000 mg GPC significantly increases plasma free choline levels (47). Numerous clinical reports suggest that GPC can improve memory and attention in patients with Alzheimer’s disease and dementia (26, 36, 48–54)

GPC advances physical and psychomotor performance in the context of muscle strength and conditioning (55–58). For instance, in a group of 13 college-aged male subjects, the administration of 600 mg GPC resulted in an increase of 98.8 N during an isometric mid-thigh pull assessment (55). Similarly, maximum velocity and maximum mechanical power were improved by the administration of 250 mg GPC (56) and nutritional supplements containing 300 mg or 150 mg GPC were shown to improve reaction time and vertical jump power (59), indicating the ergogenic properties of GPC.

The effects of GPC on cerebrovascular events remain controversial. Indeed, some investigators have conducted a multicenter clinical trial (daily intramuscular dose of 1000 mg for 28 days and oral dose of 800 mg during the following 5 months) that revealed the excellent tolerability and the therapeutic role of GPC on cognitive recovery of patients with acute stroke or transient ischemic attack (TIA) (18); on the other hand, a recent retrospective study has shown that GPC is associated with a higher 10-year incident stroke risk in a dose-response manner after adjusting for traditional cerebrovascular risk factors (60). A potential explanation for these different findings could be the diverse effects of GPC supplementation on the gut microbial community structure: in this sense, a recent preclinical report demonstrated that GPC can cause a shift in the murine microbiota, characterized by increased abundance of *Bacteroides*, *Parabacteroides*, and *Ruminococcus*, and decreased abundance of *Lactobacillus*, *Akkermansia*, and *Roseburia* (61).

Most recently, in a prospective study, GPC was suggested to enrich listening comprehension in older adults using hearing aids (62). Due to its action on the parasympathetic nervous system, GPC has also shown beneficial effects in patients with dry eye (keratoconjunctivitis sicca) and its combination with D-Panthenol accelerated and modulated the repair of the corneal innervation after cataract surgery (63–66).

## Other forms of choline supplementation

In addition to GPC, other supplements are available to ensure an adequate intake of choline (**Table 2**). One of the most used is choline bitartrate, which has shown favorable effects both in preclinical and clinical studies, especially in terms of improved cognitive function (67–73).

**Table 2:** Characteristics of the main different forms of choline supplementation.

	GPC	Choline bitartrate	Lecithin	Citicoline
**IUPAC Name**	[(2R)-2,3-dihydroxypropyl] 2-(trimethylazaniumyl)-ethyl phosphate	(2-hydroxyethyl)-trimethylazanium (2R,3R)-3-carboxy-2,3-dihydroxypropanoate	[(2R)-3-hexadecanoyloxy-2-[(9E,12E)-octadeca-9,12-dienoyl]oxypropyl] 2-(trimethylazaniumyl)-ethyl-phosphate	[[(2R,3S,4R,5R)-5-(4-amino-2-oxopyrimidin-1-yl)-3,4-dihydroxyoxolan-2-yl]methoxy-hydroxyphosphoryl] 2-(trimethylazaniumyl)-ethyl phosphate
**Molecular Formula**	C_8_H_20_NO_6_P	C_9_H_19_NO_7_	C_42_H_80_NO_8_P	C_14_H_26_N_4_O_11_P_2_
**Molecular Weight (g/mol)**	257.22	253.25	758.075	488.32
**Color/Form**	Solid	White crystalline powder	Yellow-brownish powder	White crystalline powder
**Odor**	Odorless	Odorless or faint trimethylamine-like odor	Odorless or has nut-like smell	High doses can cause fishy odor
**Taste**	No taste	Acidic taste	Nutty taste	Neutral
**Melting Point**	142.5°C	149-153°C	236.1°C	240-242°C
**Solubility**	Very soluble in water	Freely soluble in water; slightly soluble in alcohol; insoluble in ether, chloroform, and benzene	Low solubility in water, but serves as an excellent emulsifier	Very soluble in water

Importantly, a prospective randomized cross-over study was designed to compare four different choline supplements in terms of their impact on plasma concentration and kinetics of choline; participants received a single dose of 550 mg/d choline equivalent in the form of choline chloride, GPC, egg-PC, and choline bitartrate, in randomized sequence at least 1 week apart; the analysis of these revealed no difference in the area-under-curve of choline plasma concentrations after intake of the different supplements (74).

The main clinical trials assessing the effects of choline supplementation, in different formulations, are reported in **Table 3**.

**Table 3:** Main clinical studies investigating choline supplementation.

Trial	Supplementation	Subjects	Results	Ref.
Randomized cross-over study (DRKS00020454)	choline chloride, choline bitartrate, GPC, egg-PC	6 healthy adult men	-All supplements promptly raised choline and betaine levels to a similar extent, with egg-PC showing the latest peak. Considering TMAO may have unfavorable effects, egg-PC might be the best choline supplementation in adults.	(74)
Randomized double-blind placebo-controlled parallel clinical trial (IRCT20110123005670N25)	500 mg/d choline and 500 mg/d magnesium co-supplementation	96 patients with type 2 diabetes mellitus	-Combination of choline and magnesium intake have better outcomes in improving endothelial dysfunction and inflammation as compared to single supplementation alone	(75)
Randomized partially blinded single-center trial (NCT02509728)	enteral choline (30 mg/kg/day), DHA (60 mg/kg/day), or both	24 inborn preterm infants < 32-week postmenstrual age	-Co-supplementation may enhance DHA utilization. However, choline supplementation did not increase trimethylamine-N-oxide (TMAO) levels	(76)
Clinical open multicenter trial	1000 mg i.m. for 28 days and orally at the dose of 400 mg t.i.d. during the following 5 months after the first phase	2044 patients suffering from recent stroke or transient ischemic attacks	-Excellent tolerability and therapeutic role of GPC on cognitive recovery of patients with acute stroke or transient ischemic attack	(18)
Randomized, double-blind, controlled feeding study (NCT-1127022)	480 or 930 mg choline/d	29 women (≥21y) entering their 3^rd^ trimester of pregnancy, 24 eligible infants	-Infants with higher maternal choline intake demonstrated high information processing speed which lasted for at least the first year of postnatal life	(77)
Single-center, randomized, double-blind, parallel-group study (NCT03194659)	550 mg choline/d	Healthy pregnant person in their second trimester (21-40y)	-Maternal plasma choline metabolome (especially betaine) is very receptive to prenatal choline supplementation	(78)
Randomized, double-blind, placebo-controlled trial (PACTR202005864845358)	2 g of choline/d	52 infants born to heavy-drinking women who consumed choline supplementation during pregnancy	-Gestational choline supplementation alleviates alcohol exposure effects on neonatal brain volumes, choline may be neuroprotective against brain structural deficits related to prenatal alcohol exposure	(79)
Single-center, randomized, double-blind, parallel-group study (NCT03194659)	500 mg/d choline and 200 mg docosahexaenoic acid	30 pregnant women	-Prenatal choline supplementation (administered across the second and third trimesters of pregnancy) improved hepatic export of docosahexaenoic acid	(80)
Randomized, double‐blind, parallel‐group controlled trial (NCT01127022)	480 or 930 mg choline/d	Children born to women during their 3^rd^ trimester of pregnancy	-Prenatal choline supplementation enhances child sustained attention (7-year follow up)	(81)
Randomized, double‐blind, parallel‐group controlled trial (NCT01127022)	480 or 930 mg choline/d	26 healthy third-trimester pregnant women	-Maternal choline supplementation modulates biomarkers of vitamin B12 status in pregnancy	(82)
Randomized, Double-Blind, Placebo-Controlled Clinical Trial (NCT03369925)	500 mg/d citicoline	100 healthy men and women aged between 50 and 85y with age-associated memory impairment	-Regular consumption of citicoline improved attention and may be beneficial against memory loss due to aging	(83)
Randomized double-blind, placebo-controlled trial (NCT00720343)	20 g of lecithin	60 women having open gynecological surgery	-No analgesic benefit with oral choline supplementation between groups at rest or with movement.	(84)
Randomized controlled trial	500 mg and 250 mg GPC	48 healthy college-aged males	-Increased maximum velocity and maximum mechanical power	(56)
Double-blind, placebo-controlled crossover	600 mg GPC	13 healthy college-aged males	-Enhanced strength and performance especially the lower body force production	(55)
Randomized double-blind Placebo-controlled clinical trial (NCT01911299)	5.25 ml of liquid GPC (~1240 mg GPC), equivalent to 625 mg of choline	5-10y children with FASD	-General neurocognitive processes such as memory and attention, executive functioning, and hyperactivity pre- and post-intervention were not enhanced questioning the therapeutic window of choline for its efficacy	(85)
Randomized, double-blind, placebo-controlled trial (NCT01149538)	500 mg/d choline bitartrate	18 children aged 2.5-5y with FASD (after 7-year follow-up)	-Improved processing speed of lower-order executive tasks and better corpus callosum white matter microstructure and neurocognitive outcomes.	(86)
Randomized, controlled cross-over clinical trial (NCT03877003)	Three eggs/d, 400 mg/d choline as choline bitartrate	23 men and women aged 35-70y with metabolic syndrome	-Plasma lutein and zeaxanthin were increased but plasma TMAO did not elevate eggs intake or choline bitartrate supplementation for 4 weeks -no significant effects on gut microbiota	(87)
Randomized, double-blind, placebo-controlled intervention trial (ISRCTN82708510)	1 g choline per day as choline bitartrate	42 healthy postmenopausal women aged 49-71y	-Choline supplementation in postmenopausal women increases circulating free choline as well as methyl donor betaine	(88)
Placebo-controlled double-blind study	2 g of choline bitartrate	30 healthy individuals	-Enhanced visuomotor performance	(70)

## Choline bitartrate

Choline bitartrate (C_9_H_19_NO_7_) is a white crystalline powder with no odor. Its molecular weight is 253.25 g/mol with 41.1% choline (104 g/mol choline in 253.25 g/mol choline bitartrate); 2 g of choline bitartrate administration provides 800 mg of choline action (70). It is freely soluble in water, slightly soluble in alcohol and insoluble in ether, chloroform, and benzene.

Choline bitartrate is widely used in dietary supplements. One of the main advantage of bitartrate is its lower hygroscopicity (89), a feature that in the last years has triggered an increase of its use. The methyl donor betaine, a choline derivative, has been shown to facilitate the cytosolic re-methylation of homocysteine to methionine in a reaction catalyzed by the enzyme betaine-homocysteine S-methyltransferase (BHMT). The same reaction is also catalyzed by the methionine synthase, which uses methyl-cobalamin as a co-factor and is a vitamin B12 dependent enzyme (82, 90). Preclinical studies have reported a choline-sparing effect of vitamin B12 supplementation (91–93) and patients deficient in vitamin B12 have lower blood concentrations of choline (94). These aspects provide a strong rationale for the preparation of formulations in which choline, especially choline bitartrate, is associated with vitamin B12.

## Lecithin

Lecithin is a mixture of fats and can be obtained from food such as egg yolks (actually, the term lecithin derives from the Greek word λέκιθος, *lekythos*, which means ‘egg yolk’), soybeans, and nuts (95, 96). PC represents one of the main components of lecithin, albeit the two terms are sometimes used interchangeably. Lecithin is essential to cells in the human body. Since lecithin is converted into ACh, its consumption increases ACh concentrations in the brain (97). Several studies have been carried out showing the effects of consumption of lecithin on hypercholesterolemia and cardiovascular disorders (98, 99).

## Citicoline

Citicoline is a brain chemical that occurs naturally in the cells, especially organs, of human and animals. It is a natural precursor of phospholipid synthesis, chiefly PC, and serves as a source of choline in the metabolic pathways for biosynthesis of ACh in the body (100). Citicoline enhances cerebral metabolism and has neuroprotective properties in animals and humans (101–103). Citicoline is effective in facilitating cognitive improvement in various conditions, including vascular and degenerative dementias, cerebrovascular diseases, amyotrophic lateral sclerosis, Alzheimer’s disease, and also Parkinson’s disease (104, 105); indeed, citicoline increases brain dopamine levels and may inhibit dopamine reuptake (104).

## Choline supplementation and endothelial dysfunction

Endothelial cells play a crucial role in the exchange of choline and other nutrients between plasma and brain tissue (75, 106–108). Thus, choline must be incorporated into endothelial cells to be transported to the blood-brain barrier (109). Choline supplementation was shown to be effective against hypoxia-induced endothelial dysfunction by Zhang and co-workers, who demonstrated that choline enhanced rat aortic endothelial cell proliferation during hypoxia by secreting vascular endothelial growth factor (VEGF) (6). Moreover, choline supplementation activated the α7 non-neuronal nicotinic ACh receptors (nAChRs) and served as a key function in regulating blood vessels. Thus, choline can be protective against hypoxia-induced endothelial dysfunction (6, 110). Although the benefits of choline have been reported, the exact mechanisms in protecting endothelial function are yet to be fully defined.

Some investigators have reported that endothelial dysfunction is linked with various cardiovascular diseases (111, 112). Several studies have demonstrated the role of high choline intake and its metabolite trimethylamine N‐oxide (TMAO) in endothelial dysfunction and atherosclerosis (111, 113–117). Instead, phloretin, a flavonoid extracted from apple leaves, plays a protective role, and improves vascular endothelial dysfunction and liver injury (111).

Models of endothelial dysfunction like hypoxia or oxygen and glucose deprivation (OGD) were used to evaluate the effects of citicoline on human umbilical vein endothelial cells (HUVECs) and mouse brain microvascular endothelial cells (bEnd.3s) (105, 118–120). Citicoline attenuated the hypoxia/OGD-induced increase in endothelial permeability *via* upregulating the expression of tight junction proteins including zonula occludens-1, occludin, and claudin-5. Thus, citicoline could be an efficient therapeutic drug for targeting diseases characterized by endothelial barrier breakdown (105).

## Choline supplementation and cardio-metabolic disorders

Choline plays a protective role in the heart and may be a promising candidate to improve doxorubicin-induced cardiotoxicity *via* vagal activity and Nrf2/HO-1 pathway (121). Moreover, choline exhibits protective effects against cardiovascular disorders, including arrhythmias, cardiac hypertrophy, and ischemia/reperfusion (I/R)-induced vascular injury by inhibiting the ROS-mediated Ca^2+^/calmodulin-dependent protein kinase II pathway (122–124). Citicoline acts as a myocardial protector from I/R injury *via* inhibiting mitochondrial permeability transition (125). Choline was also shown to ameliorate cardiovascular damage by slowing the progression of hypertension and enhancing cardiac function in spontaneously hypertensive rats (126).

Low amounts of choline can reduce cardiovascular risks and inflammatory markers as they have lowering effect on plasma homocysteine (127). In contrast, a choline‐ or carnitine‐rich diet was reported to promote atherosclerosis in mice as it increased the formation of TMAO produced by gut microbiota-related metabolite of choline (128). Similarly, other papers have reported the association of TMAO with an increased risk of cardiovascular disease and mortality (60, 113, 129–131). Dietary lecithin has shown favorable results with potential application in the treatment of dyslipidemia associated with metabolic disorders (132). Obesity is linked with several cardio-metabolic chronic diseases, such as non-alcoholic fatty acid liver disease (NAFLD), type-2 diabetes, and cardiovascular disease. Numerous studies have also investigated the beneficial effects of lecithin on obesity-related dyslipidemia (132–135). Lecithin-rich diets have hypocholesterolemic effects and display anti-atherogenic properties (136).

Intake of choline and betaine co-supplementation was not associated to cancer or cardiovascular disease; however, an adverse cardiovascular risk factor profile was linked with high choline and low betaine levels in plasma. Therefore, choline and betaine demonstrated opposite relationships with major components of metabolic syndrome (92). Choline and betaine supplementation has not been extensively studied in clinical trials for treating obesity and maintaining normal systemic metabolism. Notwithstanding, Sivanesan and co-workers revealed that choline and betaine administration is favorable for obese and insulin resistant Pcyt2^+/-^ mice; they suggested that choline and betaine supplementations could be beneficial for the treatment of obesity and diabetes due to their participation in mitochondrial oxidative phosphorylation (137).

## Choline supplementation and cognitive dysfunction

Environmental factors may contribute to the pathological progression of neurodegenerative diseases and epilepsy. Remarkably, dietary nutrients play an important part in facilitating mechanisms related to brain function (138). As mentioned above, ACh receptors orchestrate the immune response in the central nervous system, and their dysregulation plays a part in the pathogenesis of Alzheimer’s disease (139–144). In fact, Velasquez and collaborators demonstrated that a lifelong choline supplementation may have beneficial cognitive effects such as decreasing amyloid-β plaque load and improving spatial memory in the APP/PS1 mouse model of Alzheimer’s disease. Moreover, consumption of healthy diet throughout life may reduce Alzheimer’s disease pathology (139). In another paper, the same group reported that maternal choline supplementation has profound benefits in Alzheimer’s disease pathology by reducing brain homocysteine levels across multiple generations (145). Several studies have been carried out to investigate the impact of choline supplementation on cognitive functioning in the Ts65Dn mouse model of Down syndrome; for instance, perinatal choline supplementation was reported to enhance emotion regulation in Down syndrome (146). Other studies revealed that maternal choline supplementation improves spatial learning, increases adult hippocampal neurogenesis and basal forebrain cholinergic neurons (147, 148). Bottom and colleagues demonstrated that co-supplementation of choline protects against effects of prenatal ethanol exposure in fetal alcohol spectrum disorder (FASD) offspring (149). Increasing the intake of choline may also reduce spatial memory deficits due to the exposure of chemotherapeutic agents such as cyclophosphamide and doxorubicin in cancer patients (150).

Several researchers have tested the effects of high uptake of dietary choline in elderly patients suffering from impaired memory. A cross-sectional study conducted on ~2400 elderly patients demonstrated that choline intake, defined as the combination of dietary and supplement intake, correlates with cognitive performance (151). Choline supplements in the form of lecithin and choline chloride did not significantly improve memory performance in humans although some papers have reported positive outcomes in cognitive function of animal models (152–156). However, other choline supplements such as citicoline, choline bitartrate, and GPC appear to be very promising in the treatment of elderly patients suffering from dementia (49, 52, 54, 157, 158).

## Conclusions

In summary, preclinical and clinical investigations have shown that GPC and other forms of choline supplementation have beneficial effects especially in terms of improved endothelial function and cognitive performance. Notwithstanding, further dedicated studies are warranted to compare the different effects of the currently available forms of choline supplementation.

## Author contributions

All authors listed have made a substantial, direct, and intellectual contribution to the work and approved it for publication.
